# Total cervical occlusion: A solution after cervico‐isthmic cerclage failure

**DOI:** 10.1002/ijgo.70974

**Published:** 2026-03-27

**Authors:** Déborah Couet, Anne‐Gaëlle Pourcelot, Claire Szmulevicz, Perrine Capmas, Hervé Fernandez, Élodie Debras

**Affiliations:** ^1^ Department of Gynecology and Obstetrics CHU de Bicêtre, AP‐HP, DMU2 Le Kremlin‐Bicêtre France; ^2^ University Paris‐Saclay Le Kremlin‐Bicêtre France; ^3^ CESP‐Inserm U1018 “Reproduction et Développement de l'Enfant” Le Kremlin‐Bicêtre France; ^4^ University Paris‐Saclay, UVSQ, INRAE, BREED Jouy‐en‐Josas France

**Keywords:** cervico‐isthmic cerclage, last miscarriage, spontaneous preterm birth, total cervical occlusion

## Abstract

**Objective:**

Spontaneous preterm birth is mainly caused by cervical incompetence. Cervical cerclage is the gold standard for preventing mid‐trimester losses and preterm deliveries. If cervical cerclage fails, cervico‐isthmic cerclage may be indicated. This procedure can be performed via an abdominal or vaginal approach and allows for a neonatal survival rate of around 90%. If a cervico‐isthmic cerclage fails, there is no gold standard. Total cervical occlusion (TCO) could be an option. The present study aimed to analyze the efficacy of TCO using the Saling technique in cases where cervico‐isthmic cerclage has failed.

**Methods:**

Here, we present the surgical technique and our results in a small series of five cases. This single‐center study was conducted between 2009 and 2023, involving women who underwent a TCO after the first trimester of pregnancy.

**Results:**

Five women underwent this TCO: three for late miscarriage after a cervico‐isthmic cerclage during a previous pregnancy from which no child was born, and two for threatened late miscarriage in the current pregnancy (median gestational age: 17 and 21.2 weeks). All of the women had already undergone a cervico‐isthmic cerclage.

**Conclusion:**

This occlusion was effective in prolonging pregnancy for all five women. The mean gestational age at delivery was 32.5 weeks (range: 24.1–37.2 weeks). On average, TCO prolonged pregnancy by 11.7 weeks (minimum 3, maximum 16).

## 1 INTRODUCTION

Spontaneous preterm delivery is the main cause of neonatal morbidity, mortality and, later, neurologic impairment. Preterm birth rates are stable in developed countries, at 7.5% in France and 10.5% in the USA.^1^, ^2^ There is no consensus on the definition of cervical incompetence. The most common definition is the inability of the cervix to maintain a pregnancy without uterine contractions before 37 weeks. A diagnosis of cervical insufficiency is considered after a history of pregnancy losses without uterine contractions. No test can confirm this diagnosis.^3^

Further evidence suggests that the cervix plays a more significant role than previously thought. Late miscarriage and preterm delivery include uterine contractions, early activation of the decidua, ruptured membranes, and pathologically early cervical ripening.^4^ The immunological function of the cervix and its mucus plug in preventing microbial invasion is probably essential.^5^ A shortened cervix with a concomitant loss of the protective mucus plug could lead to subclinical ascending infection.^5^ Cervical cerclage can be used to increase cervical resistance, and occlusion of the external os can be performed to retain the cervical mucus plug.^6^

Cervical cerclage is the gold standard for preventing mid‐trimester losses and preterm deliveries in cases of cervical insufficiency. According to French recommendations, prophylactic cerclage is performed at the end of the first trimester for patients who have experienced three occurrences of preterm birth or late fetal loss.^7^

There are different techniques for cervical cerclage. The McDonald technique involves placing a transvaginal purse‐string suture at the cervical junction without bladder mobilization.^8^ The Shirodkar technique involves a transvaginal purse‐string suture placed following bladder mobilization to allow insertion above the level of the cardinal ligaments.^9^ If cervical cerclage fails, a cervico‐isthmic cerclage is indicated. This procedure, also known as the Benson cerclage, was initially described by Benson and Durfee and involves the placement of a sling by laparotomy.^10^ Fernandez et al. described a vaginal approach.^11^ This procedure can also be performed laparoscopically and has been found to achieve a neonatal survival rate of around 90%.^12^

**FIGURE 1:**
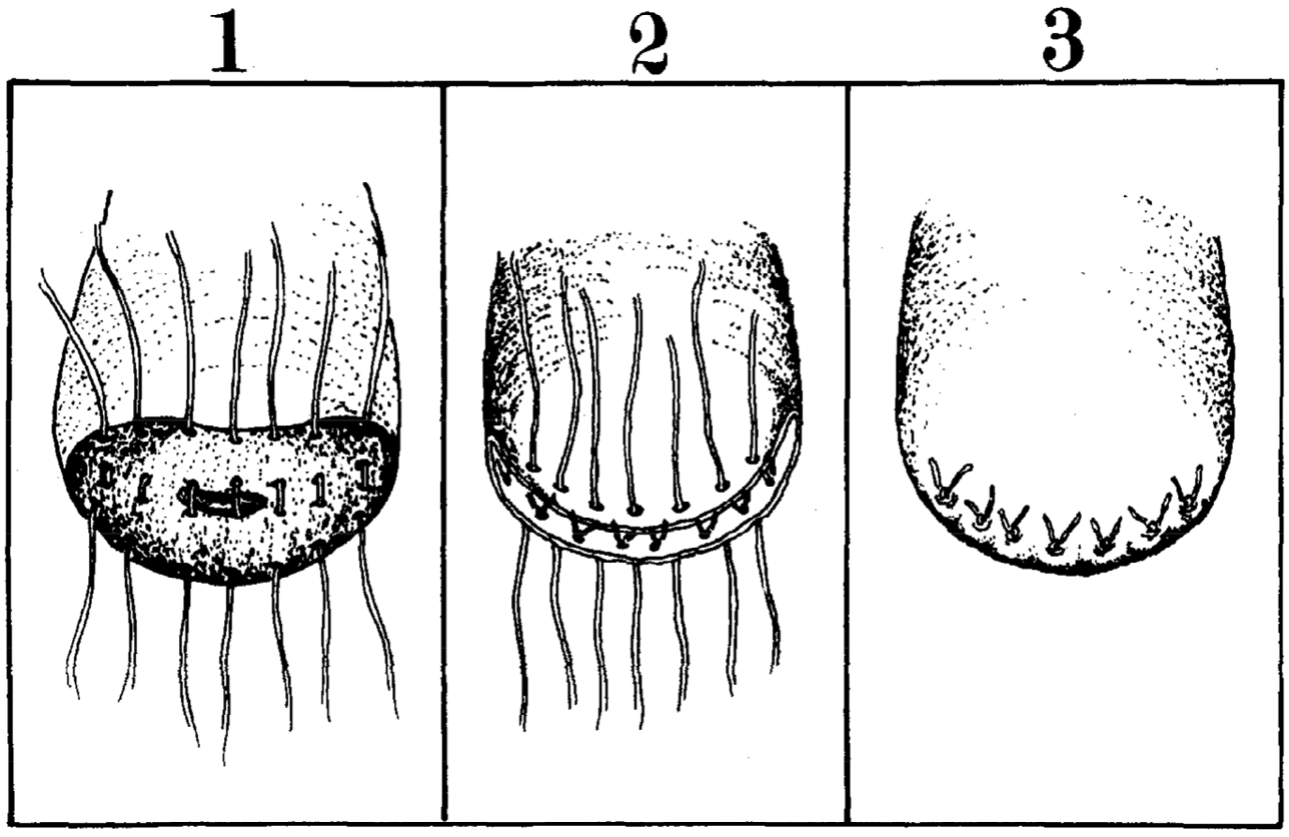
Stitching of the dissected area at the external os uteri.

**FIGURE 2:**
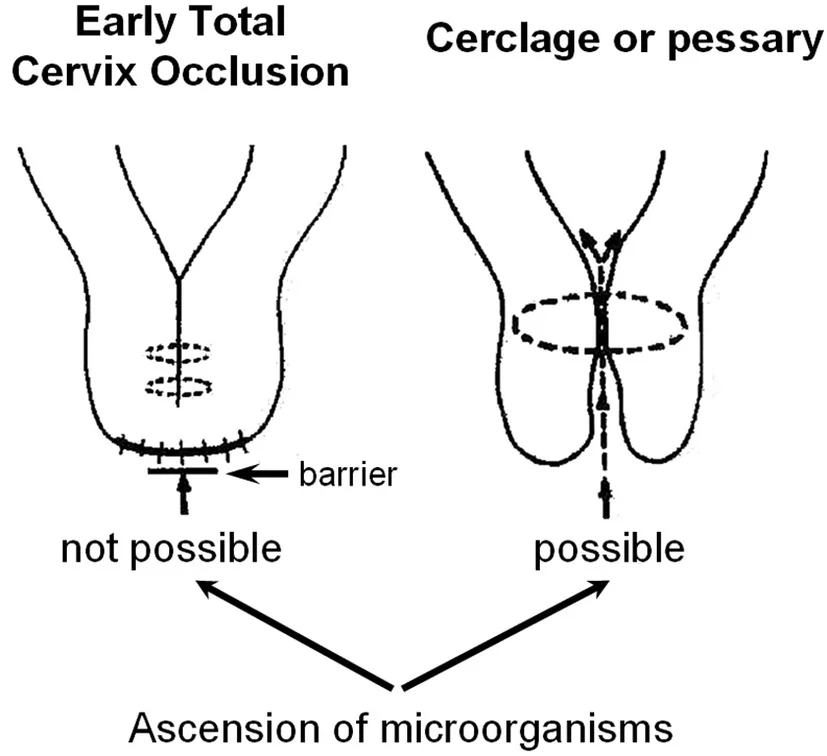
Early total cervix occlusion versus cerclage or pessary.^15^

Unfortunately, late miscarriage (cervical changes possibly associated with the presence of uterine contractions after 14 weeks of gestation and before 22 weeks of gestation) and preterm delivery (delivery before 37 weeks of gestation) can occur.^13^ We define a failure of cervico‐isthmic cerclage as either a late miscarriage or a preterm delivery before 34 weeks of gestation. There is no gold standard in case of failure of a cervico‐isthmic cerclage. One possible cause of failure is the ascent of microorganisms. Groom et al. found that the presence of visible fetal membranes at the time of the cerclage was associated with poor outcomes, with a median gestational age at delivery of 23 weeks and 4 days compared to 37 weeks and 4 days for women without visible fetal membranes (*P* = 0.002).^5^ Total cervical occlusion (TCO), which involves closing the external cervical os to prevent the ascent of opportunistic organisms, has previously been described by Saling et al.^14^ (Figure 1). Saling's occlusion increased the number of live births in women at risk of preterm birth.^15^, ^16^ (Figure 2).

The present study aimed to analyze the efficacy of cervical occlusion using the Saling technique in cases where cervico‐isthmic cerclage has failed and presents the Saling cervical occlusion technique with the aid of a Video 1. Then, the pregnancy outcomes of patients who have benefited from this cerclage are presented in a short series of five cases.

**VIDEO 1:** Surgical technique for total cervical occlusion. Video content can be viewed at https://onlinelibrary.wiley.com/doi/10.1002/ijgo.70974.

## 2 MATERIALS AND METHODS

This single center retrospective case series evaluated TCO using the Saling technique following the failure of a cervico‐isthmic cerclage. The study was conducted between January 2009 and 2025 at a French teaching hospital that serves as a referral center.

Data were collected retrospectively from all patients attending gynecologic treatment following a failed cervico‐isthmic cerclage. All patients had been referred following a cervico‐isthmic cerclage and a late miscarriage or a preterm delivery before 34 weeks of gestation.

A cervico‐isthmic cerclage failure is defined as a preterm delivery or a late miscarriage occurring despite the cerclage being in place. Cervical dilatation, protrusion through the cerclage or prelabor rupture of the membranes may occur.

The inclusion criteria were women with a single pregnancy who had received cervical occlusion due to their obstetric history, and who subsequently experienced a late miscarriage or preterm delivery. The exclusion criterion was twin pregnancy.

All patients who underwent TCO were selected from the surgical logs.

The following data were gathered: age; body mass index (BMI, calculated as weight in kilograms divided by the square of height in meters); parity; uterine abnormalities; history of cone biopsy; history of cerclage during previous pregnancies; gestational age at the time of cerclage; and pregnancy and neonatal outcomes.

Obstetric data were collected by consulting medical records retrospectively. Approval for the study was given by the ethics review committee (reference no. CEROG 2016‐GYN‐0302).

Patients gave their consent for this study, which was approved by the CEROG (2016‐GYN‐0302). They were selected retrospectively from the operative report.

The primary outcome was gestational age at delivery. The secondary outcomes were neonatal survival and surgical complications. Microsoft Excel was used to collate basic percentage data as median.

### 2.1 Surgical technique

The procedure is performed after the first‐trimester ultrasound and after the aneuploidy screening results have been obtained, if desired. A vaginal swab must be taken beforehand to treat any possible vaginal infections.

### 2.2 Materials and procedure

Two Pozzi forceps, electric scalpel with diathermic loop, multifilaments non‐absorbable threads (2/0) with a needle of 26 mm (Mersuture), claw pliers, needle holder, Mayo scissors and Breski vaginal valves are used.

The procedure is performed under locoregional (spinal) anesthesia during pregnancy if possible, or under general anesthesia if preferred by the patient, depending on their comorbidities and the anesthetist's practice. Paracetamol and nefopam are the drugs of choice for perioperative analgesia. Prophylactic antibiotic treatment with a cephalosporin is the standard practice before incision.

The patient is placed in a gynecologic position. Paracervical infiltration with physiological saline and lidocaine is performed. The procedure begins with conization using a diathermic loop to remove the epithelium from the ectocervix, the conization generation setting is as follow autocut: 3.5 and spray coagulation: 4.0. The conization should not exceed 1–2 mm in depth. Then, colporrhaphy is carried out between the anterior and posterior edges, either with two overlocks (one deep and one superficial) stitches of non‐absorbable 2/0. Alternatively, separate stitches of non‐absorbable 2/0 can be used. A non‐steroidal anti‐inflammatory intrarectal suppository is administered at the end of the procedure, repeated twice daily for 48 h to prevent uterine contractions. Level 1 analgesia is administered alongside this. Pregnancy monitoring involves monthly consultations and transvaginal ultrasounds to check for amniotic sac protrusion. No cervical measurement is required. Following TCO, patients are placed on sick leave until the cesarean section.

A cesarean section involves removing the stitches and checking the permeability of the cervical canal by passing a Hegar dilatator number 8, which is the final step in the procedure. A cesarean section is usually planned for between 37 and 38 weeks of gestation. If the patient presents signs of uterine synechiae or amenorrhea that is not due to breastfeeding we organise an office hysteroscopic to confirm the synechiae.

This surgery can trigger uterine contractions, which can result in a late miscarriage. As with any surgical procedure, there is a risk of infection, which can lead to chorioamnionitis with intact membranes. Patients with cervical changes who have undergone TCO are also at risk of premature rupture of the membranes during the surgery.

## 3 RESULTS

A total of seven cervical occlusions were performed during the study period. Of these, only five were performed after a failed cervico‐isthmic cerclage. The other two patients underwent TCO at the same time as cervico‐isthmic cerclage due to surgical difficulties caused by posterior endometriosis or a McDonald cerclage.

### 3.1 Case 1

The first patient was a 31‐year‐old woman with no living children. Despite having a McDonald prophylactic cerclage and a vaginal cervico‐isthmic cerclage, the patient had experienced late miscarriages. The cervico‐isthmic cerclage was cut during the last miscarriage. During her next pregnancy, we performed prophylactic cervical occlusion at 11 weeks and 5 days of gestation. The surgery was performed without complications under spinal anesthesia. The operative time was 16 min. She was admitted for a suspicion of chorioamnionitis at 24 weeks and 1 day of gestation and received one injection of betamethasone. She delivered the same day by cesarean section. The baby died at 8 days of a sepsis.

### 3.2 Case 2

The second patient was a 35‐year‐old woman with two children. She had a history of five late miscarriages, one vaginal delivery and one cesarean section following a cervical cerclage at 38 weeks of gestation. Despite the cerclage, she experienced a late miscarriage at 17 weeks of gestation.

At the start of her next pregnancy, we performed a prophylactic McDonald cerclage at 13 weeks. Cervical modifications were diagnosed at 21 weeks of gestation. The cervical length was 12 mm, with the membranes extending beyond the McDonald cerclage. She did not have biological inflammatory syndrome (no leukocytosis or CRP above 5). Cervical occlusion was performed without complications under spinal anesthesia. The operative time was 19 min. She delivered by cesarean section at 37.2 weeks of gestation. The baby is alive and healthy.

### 3.3 Case 3

The third patient was a 38‐year‐old woman with one child. She had a history of three late miscarriages and one cesarean section following a cervical cerclage at 38 weeks and 2 days. During the subsequent pregnancy, cervical modifications were diagnosed at 17 weeks of gestation. The cervix was open (2 cm dilatation) with no contractions. She did not have any signs of biological inflammatory syndrome. Cervical occlusion was performed without complications under spinal anesthesia. The operative time was 36 min. Intrauterine fetal demise was diagnosed at 33 weeks of gestation, after which a cesarean section was performed. Placental analysis revealed signs of chorioamnionitis.

### 3.4 Case 4

The fourth patient was a 36‐year‐old woman with no living children. She had a history of 10 late miscarriages, the last of which occurred with a cervico‐isthmic and a Shirodkar cerclage. She underwent prophylactic cervical occlusion at 14 weeks of gestation. The operative procedure took 22 min. The woman was admitted for metrorrhagia at 27 weeks of gestation. She received corticosteroid treatment to mature the lungs of the fetus. At 31.4 weeks, she presented with preterm prelabor rupture of the membranes (PPROM) and uterine contractions. She delivered by cesarean section at 31.4 weeks of gestation. The baby is alive and healthy.

### 3.5 Case 5

The fifth patient was a 37‐year‐old woman with one child. She had a history of conization of up to 22 mm for adenocarcinoma in situ. She had previously miscarried at 14 weeks of gestation without having had a cerclage and had undergone a cesarean section at 29 weeks of gestation following a cervico‐isthmic cerclage due to PPROM and suspected chorioamnionitis. She underwent prophylactic cervical occlusion at 12 weeks and 6 days of gestation. The operative time was 24 min, and the surgery was performed without complications under spinal anesthesia. She delivered by cesarean section at 36 weeks and 5 days due to her obstetric history. The baby is alive and healthy.

## 4 DISCUSSION

This study, conducted between 2009 and 2023, reports five cases of TCO among women who experienced a late miscarriage following the failure of a cervico‐isthmic cerclage during a previous or current pregnancy. The women underwent TCO after the first trimester of pregnancy. Three of the women had TCO for late miscarriage after a cervico‐isthmic cerclage during a previous pregnancy, resulting in no live births, and two had TCO for threatened late miscarriage in the current pregnancy (median gestational age: 17 and 21.2 weeks).

The mean gestational age at delivery was 32.5 weeks (range: 24.1–37.2 weeks). When TCO was performed prophylactically at the end of the first trimester, the pregnancy was prolonged by an average of 7.3 weeks beyond the maximum delivery date for the patient (minimum 3.1 weeks and maximum 11.5 weeks). In cases of threatened late miscarriage, pregnancy was prolonged by an average of 16 weeks beyond the cerclage term (minimum 16 weeks, maximum 16 weeks). On average, TCO prolonged pregnancy by 11.7 weeks (minimum 3, maximum 16). None of the women have had another pregnancy yet.

This occlusion was effective in prolonging pregnancy for four women. The fifth woman had a section of her cervical cerclage during her last pregnancy, which could explain her very early preterm birth.

Zayyan et al. presented a prospective study in which 26 patients underwent McDonald cerclage and early cervical occlusion without cervico‐isthmic cerclage. A total of 92.3% of patients delivered at term, with only one delivery occurring at 33 weeks. A total of 23.8% of patients had previously experienced failed transvaginal cerclage.^17^ The success rate of 96.2% was superior to the 89% success rate for transabdominal cerclage.^18^, ^19^

In a randomized trial of 297 patients published in 2013, Brix et al. found no significant effect of cervical occlusion with cervical cerclage in women with primary prophylactic cerclage, or in women with secondary or tertiary therapeutic cerclage. However, this study only used the McDonald or Shirodkar cerclage technique. They also used a simpler technique involving only a continuous suture and no removal of the epithelial lining, as described by Saling et al.^20^ Removing the epithelial lining allows for better occlusion due to the eroded edges.

The present study is the first to research the association between cervico‐isthmic cerclage and TCO. Cervico‐isthmic cerclage via a vaginal approach yields satisfactory results for ongoing pregnancies, with a median gestational age at birth of 37.2 weeks and an overall neonatal survival rate of 90%.^5^, ^12^, ^21^ There is no consensus on the next step following cervico‐isthmic cerclage failure. TCO should be considered after failure of a cervico‐isthmic cerclage. Furthermore, early cervical occlusion should be considered for women who do not experience cervical relief following conization or trachelectomy.

A subsequent study involving more cases and conducted in a prospective manner would be necessary.

## 5 CONCLUSION

Despite the small number of women included in the study, it shows that early cervical occlusion following cervico‐isthmic cerclage failure can prolong pregnancy. Early cervical occlusion should be a straightforward and safe option for women in the event of cervico‐isthmic cerclage failure.

## AUTHOR CONTRIBUTIONS

Déborah Couet: Corresponding author. Anne‐Gaëlle Pourcelot: Acquisition, analysis and interpretation of data. Claire Szmulevicz: Acquisition, analysis and interpretation of data. Perrine Capmas: Acquisition, analysis and interpretation of data. Hervé Fernandez: Final approval. Élodie Debras: Final approval.

## FUNDING INFORMATION

The authors have nothing to report.

## CONFLICT OF INTEREST STATEMENT

The authors report no conflict of interest.

## Data Availability

Data sharing is not applicable to this article as no new data were created or analyzed in this study.
